# Tumour necrosis factor alpha downregulates human hemojuvelin expression via a novel response element within its promoter

**DOI:** 10.1186/1423-0127-19-83

**Published:** 2012-09-21

**Authors:** Mohamed Fouda Salama, Henry K Bayele, Surjit SK Srai

**Affiliations:** 1 Department of Structural and Molecular Biology, Division of Biosciences, University College London, Gower Street, London, WC1E 6BT, UK; 2 Department of Biochemistry, Faculty of Veterinary Medicine, Mansoura University, Mansoura, Egypt

**Keywords:** Inflammation, Hemojuvelin, TNF-α

## Abstract

**Background:**

Iron homeostasis is chiefly regulated by hepcidin whose expression is tightly controlled by inflammation, iron stores, and hypoxia. Hemojuvelin (HJV) is a bone morphogenetic protein co-receptor that has been identified as a main upstream regulator of *hepcidin* expression; *HJV* mutations are associated with a severe form of iron overload (Juvenile haemochromatosis). Currently however, there is no information on how *HJV* is regulated by inflammation.

**Methods:**

To study the regulation of *Hjv* expression by inflammation and whether Hfe has a role in that regulation, control and LPS-injected wild type and *Hfe* KO mice were used. Moreover, human hepatoma cells (HuH7) were used to study the effect of IL-6 and TNF-α on *HJV* mRNA expression.

**Results:**

Here we show that LPS repressed hepatic *Hjv* and *BMPs*, while it induced *hepcidin* 1 expression in wild-type and *Hfe* KO mice with no effect on hepatic pSMAD 1, 5, 8 protein levels. In addition, exogenous TNF-α (20 ng/mL) decreased *HJV* mRNA and protein expression to 40% of control with no effect on *hepcidin* mRNA expression in 24 hours. On the other hand, IL-6 induced *hepcidin* mRNA and protein expression with no effect on *HJV* mRNA expression levels. Moreover, using the *HJV* promoter-luciferase reporter fusion construct *(HJVP1.2-luc)*, we showed that the basal luciferase activity of *HJVP1.2-luc* was inhibited by 33% following TNF-α treatment of HuH7 transfected cells suggesting that the TNF-α down-regulation is exerted at the transcriptional level. Additionally, mutation of a canonical TNF- alpha responsive element (TNFRE) within *HJVP1.2-luc* abolished TNF-α response suggesting that this TNFRE is functional.

**Conclusions:**

From these results, we conclude that TNF-α suppresses *HJV* transcription possibly via a novel TNFRE within the *HJV* promoter. In addition, the results suggest that the proposed link between inflammation and BMP-SMAD signalling is independent of HJV and BMP ligands.

## Background

Inflammatory stimuli are associated with profound alterations in iron homeostasis. Among the most crucial of these changes are the redistribution of iron into reticuloendothelial macrophages, and reduced intestinal iron absorption causing hypoferremia [1-4]. Similar findings have also been reported in mice exposed to endotoxins [5]. The impairment of iron efflux by enterocytes and reticuloendothelial macrophages is believed to play an important role in host defence against infection and cancer by limiting iron availability for pathogen or cancer cell replication [6,7]. However, one consequence of this is the anaemia of inflammation [8]. Pro-inflammatory cytokines have been shown to modulate the expression of iron transport and storage proteins in a variety of cell types [9-11]. During inflammation, IL-6 induces *hepcidin* expression through phosphorylation of Stat3 that binds to the Stat3 response element (Stat3RE) within the *hepcidin* gene promoter [12-14], increases *hepcidin* expression which in turn decreases intestinal iron absorption [15-19]. To better understand the biology of this, a study showed that humans injected with bacterial lipopolysaccharide (LPS) produced IL-6 within 3 hours, with concomitant increases in circulating hepcidin, and resulting in hypoferremia and reduced serum iron; similar findings were made in humans injected with IL-6 [20,21]. Sterile inflammation induced with turpentine produced similar responses in mice [22,23]. The finding that hepcidin is modulated by inflammatory cytokines or LPS has linked its expression levels to the anaemia of inflammation, otherwise referred to as the anaemia of chronic disease [17,24].

Hemojuvelin, a Bone Morphogenetic Protein (BMP) co-receptor, has been shown to be an important upstream regulator of *hepcidin* expression [25]. In mice lacking *Hjv*, hepcidin induction in response to iron is abrogated. On the other hand, these mice retain the ability to regulate hepcidin in response to inflammation by LPS and IL-6, though to a lesser extent than wild-type animals [26]. Moreover, *Hjv* expression was down-regulated in the liver of mice injected with LPS [26]. These findings suggest that Hjv may not be directly involved in the hepcidin response to inflammatory stimuli while it is required for hepcidin response to iron. Recently it has been shown that mice lacking hepatic Smad-4 express very low hepcidin levels. In addition, the response of hepcidin to inflammation was abrogated in these mice [27]. Although the proximity of the Stat3RE to a BMP-response element (only 6 nucleotides apart) within the human *hepcidin* promoter [28] would suggest some amount of transcriptional cross-talk between the Stat3/IL-6 [13] and the Smads/BMP signalling pathways in hepcidin induction; however, this link remains to be clarified.

Evidence for the requirement of Hfe for *hepcidin* induction during inflammation is conflicting. A group reported that mice lacking *Hfe* do not respond to LPS with increased *hepcidin* expression as wild-type animals [17]. In another study, the LPS response was significantly different among animals [29]. A recent study showed that *Hfe* knockout (KO) mice were just as able to regulate *hepcidin* in response to LPS as wild-type animals [30]. These contradictory findings on the requirement of Hfe for *hepcidin* induction during acute inflammation might be partly explained by different concentration of LPS used in these studies, but the study of *Hfe* KO mice during the inflammatory response requires further investigation. Moreover, the mechanism of regulation of *HJV* during inflammation is not clearly understood and needs further investigations. Therefore, this study aimed to understand the mechanism of HJV regulation during inflammation, to investigate the interplay between Hfe and the Smad/BMP pathway in regulating *hepcidin* expression, and to better understand how important this might be for the changes in iron flux that occur during inflammation.

## Methods

### Induction of acute inflammation in mice

Wild-type C57Bl/6 (n = 10) and *Hfe* KO (of C57Bl/6 background strain; n = 10) female mice were supplied by the Comparative Biology Unit at the Royal Free and UCL Medical School, London. All the experimental procedures were conducted in agreement with the UK animals (Scientific Procedures) Act, 1986. Animals were kept in a 12 hour light–dark cycle, provided with water ad libitum, and were fed control diet (RM1 diet; 190 mg Fe/kg diet) for six weeks after being weaned (3 weeks of age). Acute inflammation was induced by a single intra-peritoneal injection of 5 μg LPS/g body weight (Escherichia coli serotype 055:B5, Sigma, UK). Control mice were similarly injected with an equivalent volume of sterile saline solution (0.09% NaCl). The mice were terminally anaesthetised with intraperitoneal pentobarbitone sodium (Sagatal, Rhone-Merieux, UK, 90 mg/Kg) 6 hours after injection, the time at which maximal *hepcidin* induction and *Hjv* repression have been reported [26,31,32]. Livers were snap-frozen in liquid N_2_, and stored at −80°C for real-time PCR analysis, liver iron quantification, and immunoblotting.

### Cell culture and treatment with cytokines

HuH7 hepatoma cells were obtained from the UCL Institute of Hepatology. The cells were cultured in Dulbecco’s Minimal Essential Medium (DMEM; Invitrogen) and supplemented with non-essential amino acids, 10% foetal bovine serum, and antibiotics; cells were grown under standard cell culture conditions. The cells were seeded in 6-well plates (Nunc, UK). At 80-85% confluence, the medium was changed and replaced with fresh medium with vehicle (control) or with medium containing recombinant human IL-6 (10 ng/mL; R&D Systems, UK) or recombinant human TNF-α (20 ng/mL; R&D Systems). Both cytokines were reconstituted in sterile PBS containing 0.1% bovine serum albumin. After treatment, medium was removed and cells were washed with PBS.

### RNA extraction and RT-PCR

RNA was extracted from HuH7 cells and from mouse livers using TRIzol (Invitrogen) according to the manufacturer’s instructions. Total RNA (1 μg) was reverse-transcribed using the Verso cDNA kit (Thermofisher Scientific) as instructed by the manufacturer. RT-PCR was performed using Lightcycler (Roche); human *GAPDH* and mouse *β-actin* were used as internal controls. Duplicate PCR reactions were run for each target gene including *GAPDH or β-actin*; each reaction mix contained 1μL of cDNA template, 5pmol each of forward and 5pmol reverse primers, 10 μL 2× QuantiTect SYBR**®** Green PCR master mix and water to a final volume of 20 μL. As a negative control, samples without cDNA were included. The primers used for real-time PCR were synthesised by Sigma-Genosys Ltd. (Poole, UK) and are shown in (Table 1). Gene expressions were normalized to that of the *GAPDH* or *β-actin* and represented as ΔCt values. For each sample the mean of the ΔCt values was calculated.

**Table 1:** Human and mouse primers used for real-time PCR

** Primer**	**Forward (5′-----------3′)**	**Reverse (5′-----------3′)**
**human *hepcidin***	CTGCAACCCCAGGACAGAG	GGAATAAATAAGGAAGGGAGG
**human *HJV***	GGAGCTTGGCCTCTACTGGA	ATGGTGAGCTTCCGGGTG
**human *GAPDH***	TGGTATCGTGGAAGGACTC	AGTAGAGGCAGGGATGATG
**mouse *hepcidin1***	CCTATCTCCATCAACAGATG	AACAGATACCACACTGGGAA
**mouse*Hjv***	TGCCAGAAGGCTGTGTAAGG	TCTAAATCCGTCAAGAAGACTCG
**mouse*TNF-a***	CCAGACCCTCACACTCAGATCA	CACTTGGTGGTTTGCTACGAC
**mouse*IL-6***	AGTTGCCTTCTTGGGACTGA	TCCACGATTTCCCAGAGAAC
**mouse*BMP-2***	TGGAAGTGGCCCATTTAGAG	TGACGCTTTTCTCGTTTGTG
**mouse*BMP-4***	ACGTAGTCCCAAGCATCACC	TCAGTTCAGTGGGGACACAA
**mouse*BMP-6***	ATGGCAGGACTGGATCATTGC	CCATCACAGTAGTTGGCAGCG
**mouse*TGF- β***	CACCGGAGAGCCCTGGATA	TGTACAGCTGCCGCACACA
**mouse *β-actin***	GACGGCCAAGTCATCACTATT	CCACAGGATTCCATACCCAAGA

### Immunoblotting

To investigate the effect cytokines on *HJV* or *hepcidin* expression, HuH7 cells were either untreated or treated with TNF-α or IL-6 respectively for 24 hours. Proteins were extracted with RIPA buffer (Santa Cruz) containing protease and phosphatase inhibitors (Sigma); protein content was determined with the Pierce BCA protein assay system. Approximately 50 μg aliquots of homogenates were diluted with an equal volume of 2× Laemmli sample buffer, heated at 40°C for 30 min and resolved on a 12% polyacrylamide gel (Bio-Rad) at 40 mA. Proteins were transferred onto PVDF membranes (Bio-Rad), using a Bio-Rad Trans-Blot SD semi-dry blotter. After transfer, the blots were washed in distilled water (3 × 1 min) and incubated in 10% glutaraldehyde overnight. After washing 3 × 5 min with distilled water, the membrane was incubated with 5% non-fat dry milk in PBS-T (PBS/0.1% Tween 20) overnight at 4°C, with gentle agitation. A 1:2000 or 1: 1000 dilution of affinity-purified anti-hepcidin antibody [33] or anti-HJV antibody (Santa Cruz Biotechnology, UK), respectively was added and then incubated overnight at 4°C. Membrane blots were then washed 1 × 15 min and then a further 3 × 5 min with PBS-T, and primary antibodies were probed with an HRP-conjugated secondary IgG at a 1:5000 dilution in PBS-T for 1 hour at room temperature, with continuous rocking. Following secondary antibody incubation, the membrane was washed 3 × 10mins with PBS-T for at room temperature. Detection was performed with the ECL detection kit (Amersham Life Science), in a Fluor-S MultiImager (Bio-Rad). The membrane was also probed with a 1:20,000 dilution of HRP-conjugated anti-actin antibody AC-15 (Abcam) to ascertain equivalent sample loading. Hepatic expression of phosphorylated Smad-1/5/8 in saline and LPS injected mice was determined by Western blotting using a 1:500 dilution of a phospho-Smad antibody (Cell signalling, UK).

### Generation of HJV promoter-reporter construct

A portion of the human *HJV* promoter extending 1.2Kb upstream of the beginning of the first *HJV* exon was amplified by PCR from placental genomic DNA using the PTC-100 thermocycler (MJ Research) and Phusion High- Fidelity DNA Polymerase (New England Biolabs, UK). The following primers were used: sense 5^′^ CATGCTAGCAAGTGACCCTCCTGCCTCAG, the *Nhe*I restriction site is underlined; antisense 5^′^ CATCTCGAGCTGCTGTCTCACTGAGGTCA, the *Xho*I restriction site is underlined; both were synthesised by Sigma-Genosys Ltd. The PCR reaction mix contained 250 ng of human genomic DNA, forward and reverse primers (final concentration of 0.5 μM), 10μL 5× Phusion HF Buffer, 500 μM each of dNTPs, 1 unit of Phusion DNA polymerase, and water to a final volume of 50μL. The cycling parameters were 98°C for 10 seconds (denaturation), 72°C for 2 min (annealing and extension); 35 cycles of PCR were performed with a final extension for 7 min at 72°C. The PCR products were digested with *Nhe*I-*Xho*I (New England Biolabs, UK), purified with Geneclean (BIO101, UK), ligated into *Nhe*I-*Xho*I-restricted pGL3 Basic vector (Promega) using the Quick ligation kit (NEB), and transformed into DH5α competent cells (Invitrogen). Plasmids were purified using Nucleospin plasmid kit (Macherey-Nagel, Germany), and restricted with *Nhe*I and *Xho*I to identify recombinants. The promoter clone generated, *HJVP1.2-luc*, was sequenced for verification.

### Site-directed mutagenesis of HJVP1.2-luc TNF-α response element (TNFRE)

A single putative TNFRE, GGC (A/T) GCC, was visually identified in *HJVP1.2-luc*. To test whether the identified TNFRE was functional, *HJVP1.2-luc* was subjected to site-directed mutagenesis using the QuikChange Site-Directed Mutagenesis kit (Stratagene, UK) as instructed by the manufacturer. The mutant primers were as follows: sense TGAAAATGCTGGAGGaAttCTTGGAGTAGGCAGTG and antisense CACTGCCTACTCCAAGaaTtCCTCCAGCATTTTCA; mutated nucleotides (lower case) formed an *EcoR*I restriction site (underlined) that was used as a marker for successful mutagenesis of *HJVP1.2-luc*. After initial denaturation for 1 minute at 95°C, PCR cycling parameters were 95°C (1 minute), 55°C (1 minute), and 65°C (6 minutes), for a total of 18 cycles. Following *DpnI* digestion of wild-type *HJVP1.2-luc*, transformation of INVαF’ competent cells (Invitrogen) with the mutagenesis reaction and selection on LB agar/ampicillin plates, plasmid DNA was purified from overnight cultures of single colonies and digested with *EcoR*I (New England Biolabs), to distinguish wild-type from mutant *HJVP1.2-luc* The mutant construct was sequenced for authenticity and was designated *mtHJVP1.2-luc*.

### Transfections and reporter assays

HuH7 cells were seeded in 24-well plates (Corning) and transfected at ~80-90% confluence with 200 ng of either *HJVP1.2-luc* or *mtHJVP1.2-luc* using Lipofectamine 2000 (Invitrogen); reporter assays were measured and luciferase levels were normalized with respect to β-galactosidase activity as described previously [34].

## Results

### Correlative changes in iron regulatory protein expression during LPS-induced acute inflammation in vivo

**Figure 1:**
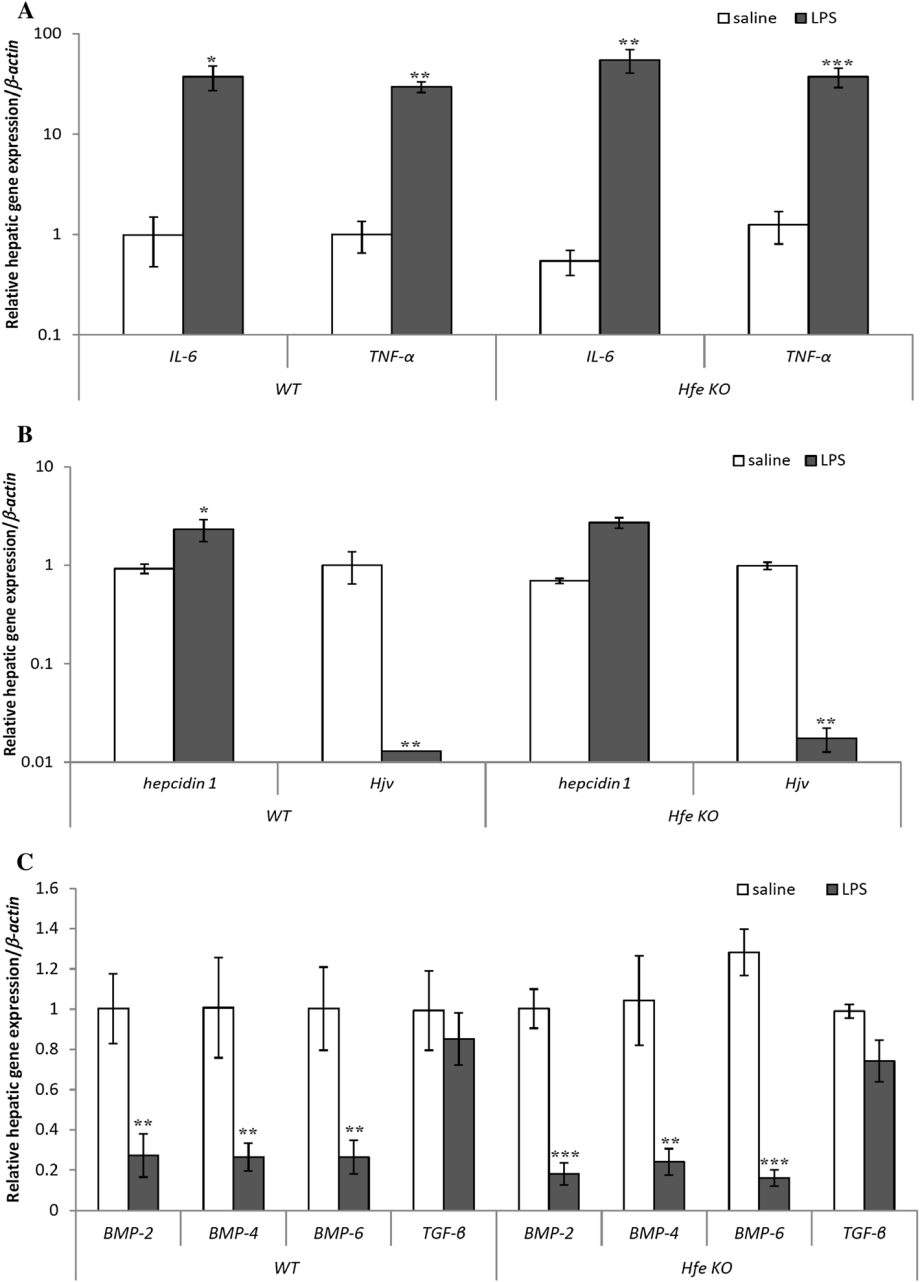
**Effect of acute inflammation on hepatic gene expression in wild-type and *Hfe KO*mice.A**) *IL-6* and *TNF-α* mRNA expression levels were analysed by real time PCR. **B**) *Hepcidin 1* and *Hjv* mRNA levels were analysed by RT-PCR. **C**) *BMP-2, BMP-4, BMP-6, and TGF-β* mRNA expression were analysed by RT-PCR. Data are mean ± SEM (n = 5).*p <0.05, **p <0.01, ***p <0.001 in LPS-injected mice compared to saline-injected controls.

Since the premise of our study was to examine how inflammation might cause changes in iron flux, we treated wild-type C57Bl/6 and *Hfe* KO mice for 6 hours with LPS to induce an acute inflammatory response. We first measured liver mRNA levels of *IL-6* and *TNF-α* in saline- and LPS- injected mice by RT-PCR. As expected, treatment with LPS significantly increased *IL-6* and *TNF-α* expression levels in the liver of wild-type and *Hfe* KO mice (Figure 1A). Since these cytokines impact *hepcidin* and *Hjv* expression respectively, we determined their mRNA levels as well as those of *hepcidin and Hjv* in saline- and LPS-injected wild-type and *Hfe KO* mice. Treatment with LPS significantly increased *hepcidin 1* expression but decreased *Hjv* expression levels in wild-type and *Hfe KO* mice; their expression profiles were similar in both genotypes (Figure 1B). Moreover, *Hfe KO* mice showed less *hepcidin 1* expression than wild-type animals although this was not statistically significant.

**Figure 2:**
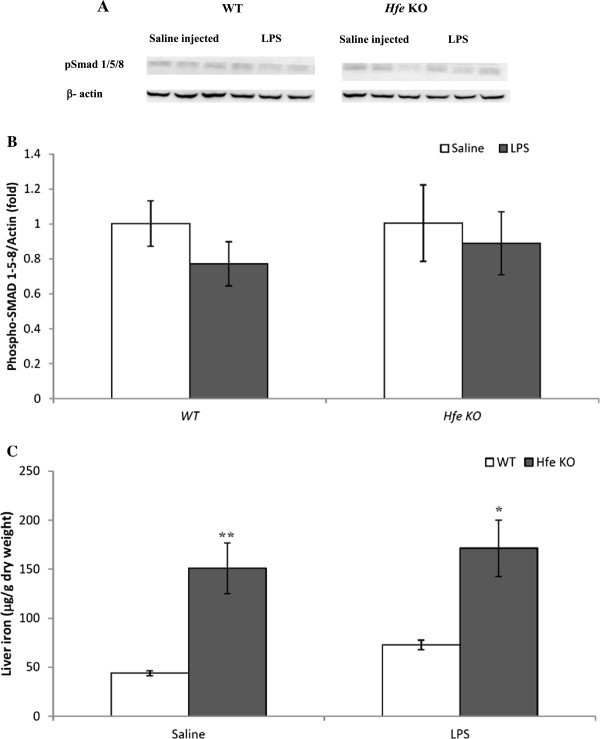
**Effect of inflammation on Smad1/5/8 phosphorylation and liver iron in wild-type C57BL/6 and *Hfe*KO mice.** (**A**) Western blotting of liver lysates from saline- or LPS-injected WT and *Hfe* KO mice using an antibody to phosphorylated Smad1/5/8; β-actin was used as loading control. (**B**) Chemiluminescence was quantified using Quantity One software to calculate the ratio of phosphorylated Smad1/5/8 to β-actin. Mean ratios of four samples (± SEM) are represented relative to the mean ratio of the saline-injected WT or *Hfe* KO mice. **C**) Liver iron was quantified; iron levels are expressed as μg/g dry weight. Data are mean ± SEM (n = 5).* denotes significant difference from LPS-injected WT mice (p <0.05). ** denotes significant difference from the saline–injected WT mice (p <0.01).

To assess if there was a link between this inflammatory response and the BMP-Smad signalling pathway and whether Hfe might be involved, *BMP 2, 4, and 6*, and *TGF-β* gene expression levels were also analysed. LPS treatment significantly decreased the mRNA expression levels of *BMP2, BMP4, and BMP6* (Figure 1C) in wild-type and *Hfe* KO mice. However, it had no effect on the expression levels of *TGF-β* and pSmad 1, 5, and 8 protein expression levels in either genotypes (Figure 2A).

Next, we asked whether these changes in iron regulatory gene expression affected liver iron levels. Liver iron content measurement showed that acute inflammation increased hepatic iron content in wild-type mice though not significantly, but there was no change in liver iron in LPS-injected *Hfe* KO mice which already showed relatively higher liver iron levels compared with both untreated or LPS-treated wild-type animals (Figure 2C).

### IL-6 induces hepcidin while TNF-α suppresses HJV expression

**Figure 3:**
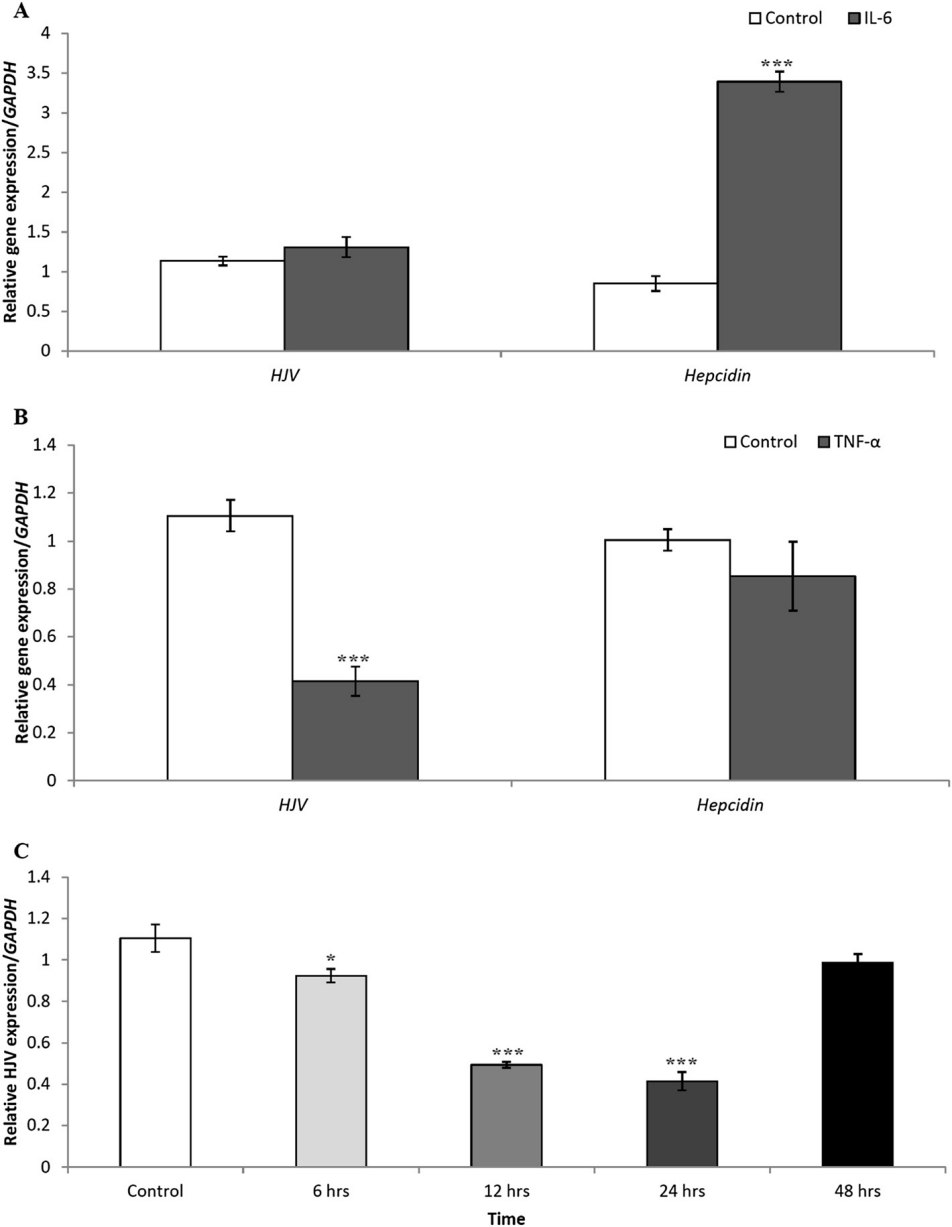
**Quantitative RT-PCR analysis of *hepcidin*and *HJV*mRNA expression in HuH7 cells following IL-6 and TNF-α treatment.A**) HuH7 cells were incubated for 24 hours in the presence of vehicle (control) or IL-6 (10 ng/mL). **B**) HuH7 cells were incubated for 24 hours in the presence of vehicle (control) or TNF-α (20 ng/mL) and quantitative PCR was performed for *hepcidin* and *HJV* gene expression. Data are mean ± SEM of 6 samples from 3 separate experiments performed in duplicate. ***p <0.001. (**C**) HuH7 cells were incubated in the presence of vehicle (control) or TNF-α (20 ng/mL) for 6, 12, 24, and 48 hours. Quantitative PCR for *HJV* mRNA expression was performed for 6 samples, each run in triplicates, in 2 separate experiments. Data are mean ± SEM . *p <0.05, ***p <0.001.

**Figure 4:**
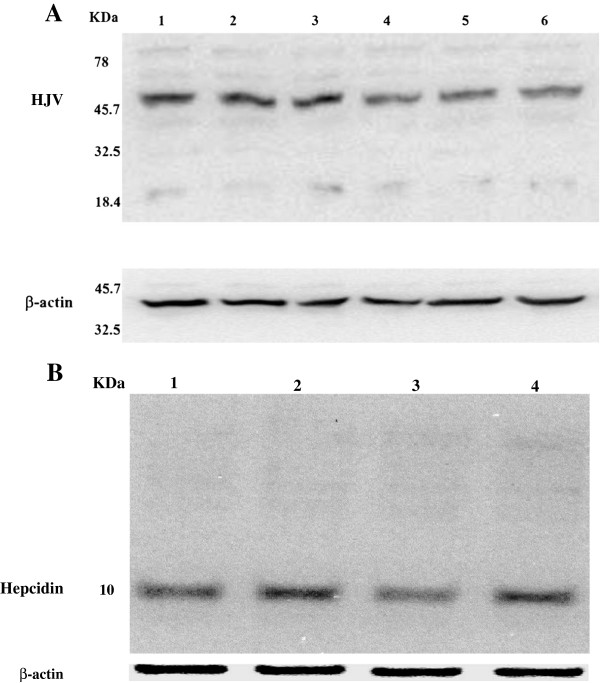
**Western blot analysis of *hepcidin*and *HJV*protein expression in HuH7 cells following IL-6 and TNF-α treatment.A**) Cell lysates from control (lanes 1-3) and TNF-α treated (lanes 4-6) cells were analyzed by Western blotting with an antibody to HJV; β-actin was used as loading control. A representative experiment is shown. (**B**) Cell lysates from control (lanes 1, 3) and IL-6 treated (lanes 2, 4) cells were analyzed by Western blotting with an antibody to hepcidin; β-actin was used as loading control. A representative experiment is shown. Protein molecular weight markers are in kDa.

To compare the expression profiles of *hepcidin* and *HJV* during the inflammatory response, we treated HuH7 cells with the pro-inflammatory cytokines, IL-6 and TNF-α for 24 hours. We found that IL-6 induced a significant increase in *hepcidin* mRNA (up to 3-fold) but it had no effect on *HJV* expression (Figure 3A). On the other hand, TNF-α significantly decreased *HJV* mRNA expression levels in a time-dependent manner but showed no discernible effect on *hepcidin* mRNA expression (Figure 3B). Maximal repression was observed after 24 hours, and *HJV* mRNA expression levels returned to normal homeostatic levels after 48 hours (Figure 3C). Next, we asked if the changes in *hepcidin* and *HJV* mRNA were reflected in their cognate protein levels. Using immunoblotting, we indeed found that hepcidin protein levels were induced in IL-6 treated cells, while HJV protein levels were significantly reduced in TNF-α treated cells compared to control (Figure 4A&B).

### TNF-α represses HJV transcription

**Figure 5:**
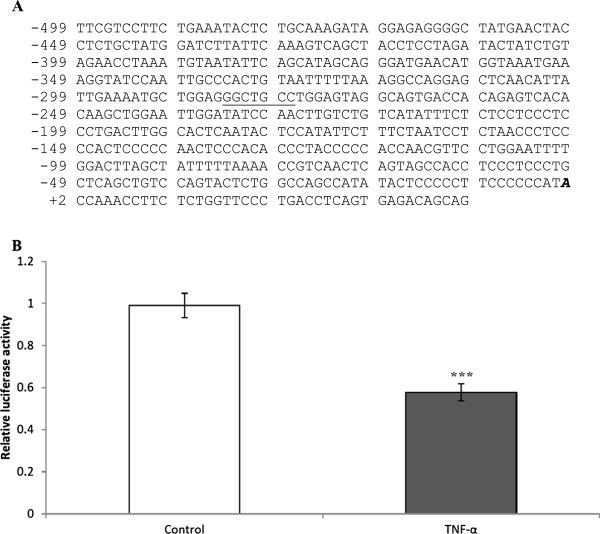
**Genomic context of the TNFRE in the *HJV*promoter; and luciferase reporter activity of *HJVP1.2-luc*transfected HuH7 cells following TNF-α treatment. A)** The consensus TNF-α RE (underlined) lies 277 nucleotides upstream of the transcription start site (bold italic). (**B**) HuH7 cells were transfected with *HJVP1.2-luc* and incubated for 24 hours with TNF-α (20 ng/ml). Luciferase expression in both treated and untreated cells were normalized to β-galactosidase expression. Data are mean ± SEM, n = 12 from 3 separate experiments; **p <0.0001.

**Figure 6:**
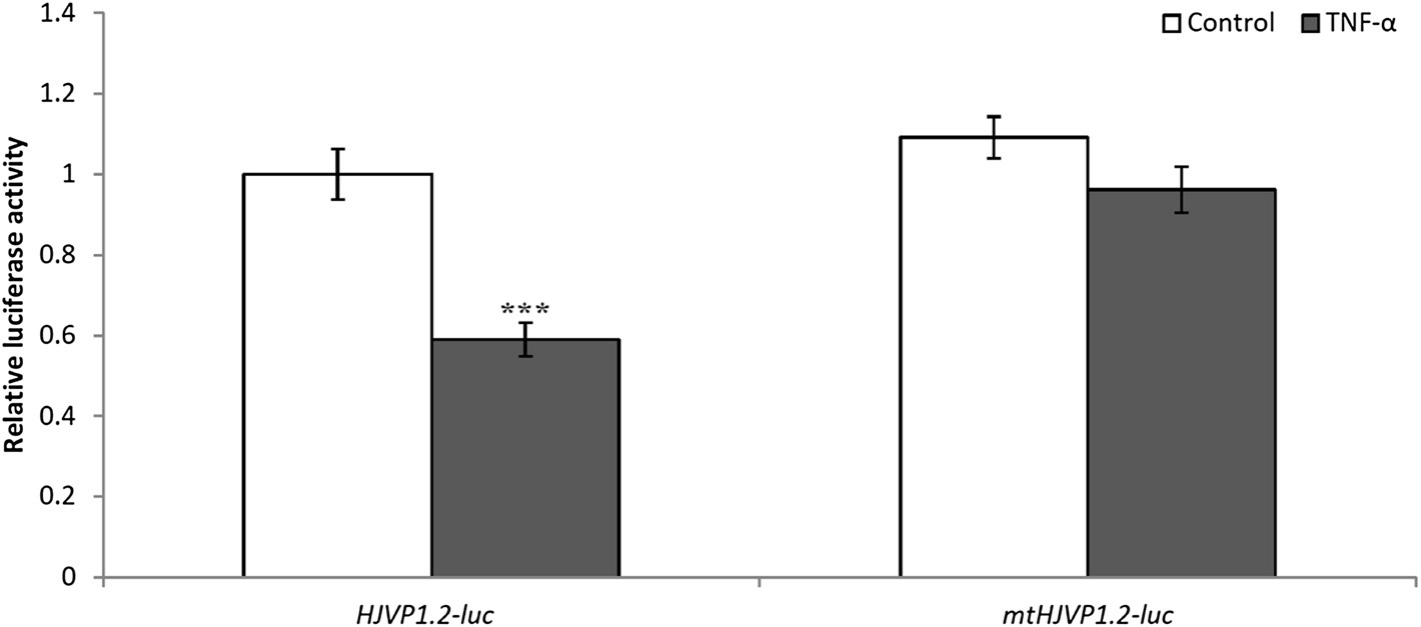
**Luciferase reporter activity of *HJVP1.2-luc*and mt *HJVP1.2-luc*transfected HuH7 cells following TNF-α treatment.** HuH7 cells were transfected with *HJVP1.2-luc* or mt*HJVP1.2-luc* to verify that the identified TNFRE is functional. Transfected HuH7 cells were incubated for 24 hours in the presence of TNF-α (20 ng/ml). Data are mean ± SEM, n = 12 of 3 independent experiments. ***p <0.0001.

We identified a single, putative TNF-α response element (TNFRE) in the *HJV* promoter, 277 nucleotides upstream of the transcription start site (Figure 5A). Similar sequences have been shown to mediate the down-regulation of several human genes in response to TNF-α including *osteocalcin*, *thrombomodulin*, *alkaline phosphatase*, and *c-myc*[35-38]. To test if a similar mechanism might underlie *HJV* regulation, we transfected HuH7 cells with *HJVP1.2-luc*. Luciferase assays showed that the basal luciferase activity of *HJVP1.2-luc* was inhibited by about 40% following TNF-α treatment (Figure 5B), suggesting that the cloned promoter region was responsive to TNF-α. In order to ascertain whether this site was functional and responsible for TNF-α suppression of *HJV* expression, it was deleted by site-directed mutagenesis to generate mt*HJVP1.2-luc*. This approach confirmed that the TNFRE alone within *HJVP1.2-luc* was sufficient for *HJV* down-regulation by TNF-α because its deletion abolished TNF-α responsiveness whereas the wild-type promoter was repressed by this cytokine (Figure 6). These data strongly suggest that TNF-α suppresses the transcription of *HJV* through this RE.

## Discussion

The thrust of our study was to gain some understanding of how inflammation might induce changes in the expression of genes that regulate iron metabolism, and to evaluate and extrapolate some possible consequences that might have in human disease. First, we investigated *hepcidin1* and *Hjv* expression in C57Bl/6 mice during acute inflammation. The results showed that LPS induced *hepcidin 1* expression in mice in accordance with previous reports [30,39], but decreased the expression of *Hjv, BMP-2, BMP-4, and BMP-6* in the liver. Decreased expression of *Hjv* by LPS is in agreement with previous reports [32,40]. Hfe did not appear to contribute to the regulation of these genes during inflammation because there were no differences in *hepcidin1* expression between wild-type and knockout mice; this is consistent with other findings [29,30] but is in sharp contrast to other reports where inflammation induced with a lower dose of LPS than the one used in this study reported blunted expression of *hepcidin* in *Hfe* KO mice [17].

The direct regulation of *hepcidin* and *HJV* expression by pro-inflammatory cytokines was also examined *in vitro* using a human hepatoma cell line (HuH7). These cells were also used to study the mechanism of *HJV* regulation during inflammation using a *HJV* promoter-reporter construct. We found that TNF-α, but not IL-6, mediated *HJV* down-regulation during inflammation, whereas IL-6 was required to induce *hepcidin* expression in HuH7 cells. Hepatic *Hjv* expression was also down-regulated by LPS in wild-type and *Hfe* KO mice, consistent with other reports [40]. These data imply that the ability to down-regulate *Hjv* expression during the acute phase response remains intact even in the absence of functional Hfe. Our results also showed that TNF-α-induced *HJV* suppression was mediated by a TNF-α response element within the *HJV* promoter.

It is well established that BMPs induce *hepcidin* expression through Smad signalling via a common mediator, Smad-4 [25,41]. However, in mice with targeted disruption of *Smad-4* in the liver, the response of hepcidin to inflammatory stimulation was blunted [41]. Moreover, a recently identified BMP-responsive element within the *hepcidin* gene promoter was found to be not only important for the BMP response but also for IL-6 responsiveness [28]. These data suggest that there might be cross-talk between the IL-6 and the BMP-Smad signalling pathways. We therefore investigated the possible interaction between inflammation and BMP/Smad signalling in LPS-induced acute inflammation in wild-type and *Hfe* KO mice. Because BMPs transmit signals through phosphorylation of Smad1, Smad5 and Smad8, the relative abundance of the phosphorylated forms of these three Smads was also quantified in liver extracts of LPS- and saline- injected wild-type and *Hfe* KO mice by Western blot analysis. Interestingly, we found that *BMP-2, BMP-4, and BMP-6* mRNA expression levels were down-regulated during acute inflammation in wild-type and *Hfe* KO mice but there was no change in phosphorylated Smad1/5/8 expression in the liver. In addition, no change was observed in *TGF-β* expression in response to inflammation that could maintain the basal expression levels of phosphorylated Smads [42]. These findings suggest that BMPs are not required for *hepcidin* induction during inflammation; we propose that if there is any link between inflammation and Smad signalling, it probably lies downstream of BMP ligands, and is probably Hfe-independent. To our knowledge, this is the first demonstration that *BMP* expression may be influenced by inflammation *in vivo*; however, the exact mechanism and its implication need further investigation.

LPS is recognized by Toll-like receptor (TLR) 4 which, upon activation, produces pro-inflammatory cytokines such as TNF-α, IL-1, and IL-6 through NFκB [43,44]. Given this information, it is unclear why the same ligand (LPS) or signalling pathway produces cytokines that have variable effects on *hepcidin* expression, i.e. that IL-6, but not TNF-α, induces *hepcidin* during inflammation. Our observation that TNF-α repressed *HJV* in liver cells strongly suggests that these cytokines impact hepcidin and iron regulation in very different ways. This TNF-α-mediated down-regulation of *HJV* expression is not only in accord with other previous findings [40] but also extends them with (our) new data which show that TNF-α uniquely drives *HJV* repression through a response element in the promoter of this gene. Mutational analysis confirmed that this element was indeed necessary and sufficient for TNF-α responsiveness. An added complication is the role of the BMPs in modulating *hepcidin* expression through HJV and at present it is unclear how the interplay between TNF-α on one hand and the BMPs on the other, affects *hepcidin* expression through HJV. However, this conundrum appears to have been resolved by recent findings that mice lacking either *Hjv* or *Bmp-6* retained the ability to induce *hepcidin* in response to inflammatory stimuli [26,45]; this indicates that neither Hjv nor BMP-6 is required for hepcidin response to inflammation. However, both Hjv and BMP-6 have been shown to be required for iron sensing by hepcidin.

**Figure 7:**
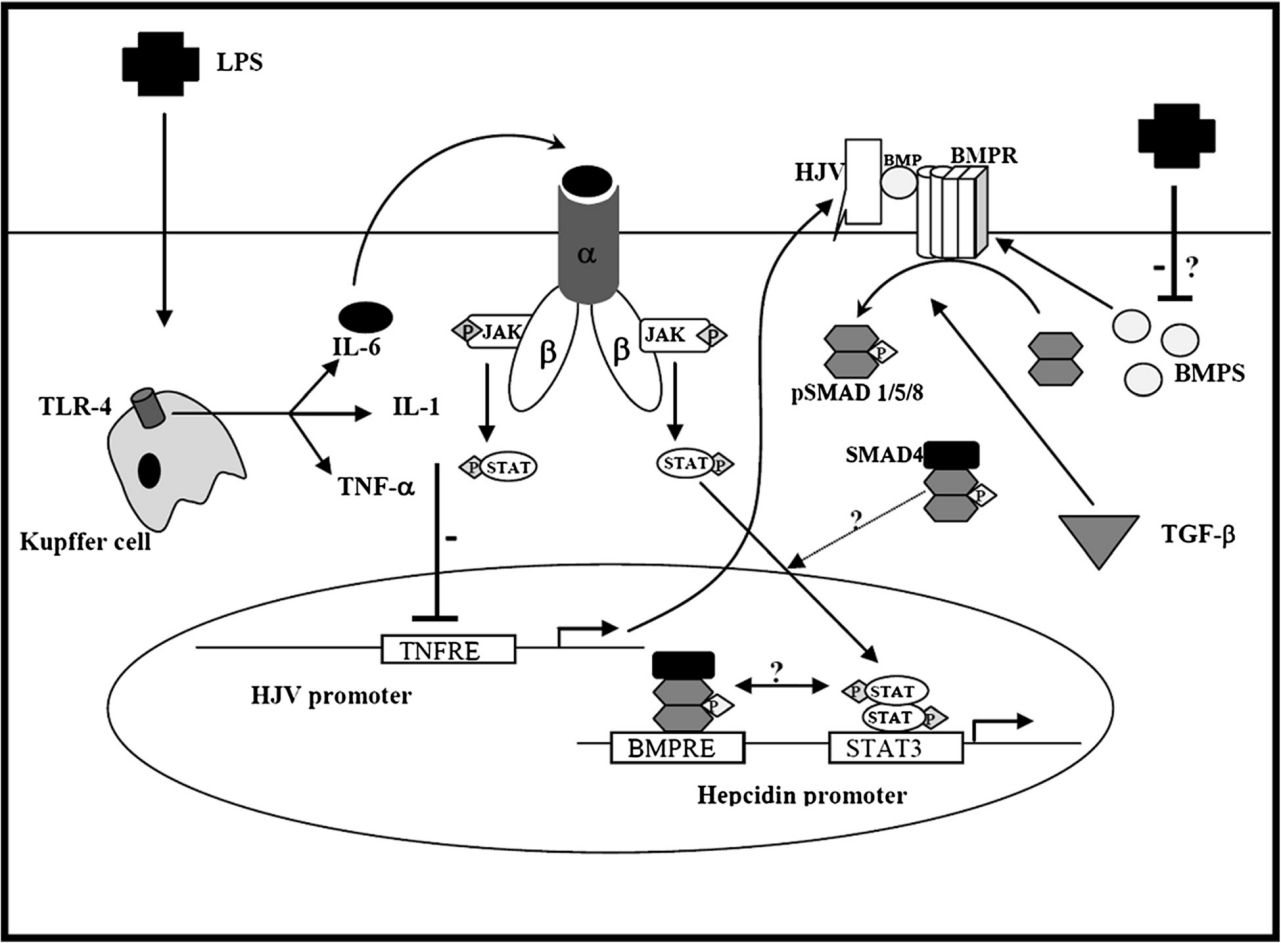
**Proposed mechanism of the inter-relationship between *hepcidin*and *HJV*expression during the inflammatory response, and the effect on iron flux.** LPS is taken up by Kupffer cells via TLR-4 and induces TNF-α, IL-1, and IL-6. TNF-α suppresses *HJV* expression via a TNFRE within the *HJV* promoter, while IL-6 induces *hepcidin* expression via the STAT3 pathway. LPS also represses the expression of *BMPs* (2, 4, and 6), via an as yet unknown mechanism. Thus, iron sensing by the BMPs, HJV and BMP receptor involved in regulating hepcidin is abrogated whereas inflammatory signalling by IL-6 through hepcidin remains intact.

On the basis of our observations we propose a mechanism (Figure 7) in which iron flux through HJV-BMP-Smad signalling is curtailed by LPS-induced TNF-α. The fundamental reason (see below) for such a control is not only interesting in itself but also because TNF-α is a first-responder, its expression is transient (being maximal at about 2 hours after LPS treatment), and returning to basal levels by 6 hours [46]; the latter time-point coincides with maximal acute phase response by the liver when *hepcidin* is most expressed. Since soluble HJV is a potent repressor of *hepcidin* expression [41], it would therefore appear that TNF-α constrains *HJV* expression in order to enable subsequent *hepcidin* induction in the acute phase response through IL-6/Stat3 signalling. In other words, TNF-α simply serves as an initiator and to prime *hepcidin* expression in the acute phase response through IL1β and IL-6 which are integral to that process. Our model is supported by recent observations that mice lacking *Bmp-6*[47] which signals through Hjv, still retained the ability to express *hepcidin* in response to LPS precisely because the early response phase had been by-passed or because the BMP-6 and IL-6 pathways are separable but convergent, i.e. there may be cross-talk between these two disparate pathways [28]. Taken together with other findings, our results may improve our understanding of how this cross-talk (or lack of it) may determine how *hepcidin* expression is regulated by infection/inflammation [48]. However, how that may determine the development and course of the anaemia that results, and the search for small-molecule antagonists that target this pathway needs further investigation.

## Conclusion

Our results demonstrate that TNF-α suppresses *HJV* transcription via a novel TNFRE within its promoter. We propose that the link between inflammation and BMP-SMAD signalling is downstream of HJV and BMP ligands; this link and its implication need further studies.

## Competing interests

The authors declare no competing interests.

## Authors’ contribution

MFS designed, performed experiments and wrote the manuscript. SKS designed experiments and edited the manuscript. HKB critically reviewed and approved the manuscript. All authors read and approved the final manuscript.
